# Quantitative network mapping of the human kinome interactome reveals new clues for rational kinase inhibitor discovery and individualized cancer therapy

**DOI:** 10.18632/oncotarget.1984

**Published:** 2014-05-18

**Authors:** Feixiong Cheng, Peilin Jia, Quan Wang, Zhongming Zhao

**Affiliations:** ^1^ Department of Biomedical Informatics, Vanderbilt University School of Medicine, Nashville, Tennessee, USA; ^2^ Center for Quantitative Sciences, Vanderbilt University Medical Center, Nashville, Tennessee, USA; ^3^ Department of Cancer Biology, Vanderbilt University School of Medicine, Nashville, Tennessee, USA; ^4^ Department of Psychiatry, Vanderbilt University School of Medicine, Nashville, Tennessee, USA

**Keywords:** Kinome, kinase-substrate interaction, phosphorylation, interactome, resistance, systems biology

## Abstract

The human kinome is gaining importance through its promising cancer therapeutic targets, yet no general model to address the kinase inhibitor resistance has emerged. Here, we constructed a systems biology-based framework to catalogue the human kinome, including 538 kinase genes, in the broader context of the human interactome. Specifically, we constructed three networks: a kinase-substrate interaction network containing 7,346 pairs connecting 379 kinases to 36,576 phosphorylation sites in 1,961 substrates, a protein-protein interaction network (PPIN) containing 92,699 pairs, and an atomic resolution PPIN containing 4,278 pairs. We identified the conserved regulatory phosphorylation motifs (e.g., Ser/Thr-Pro) using a sequence logo analysis. We found the typical anticancer target selection strategy that uses network hubs as drug targets, might lead to a high adverse drug reaction risk. Furthermore, we found the distinct network centrality of kinases creates a high anticancer drug resistance risk by feedback or crosstalk mechanisms within cellular networks. This notion is supported by the systematic network and pathway analyses that anticancer drug resistance genes are significantly enriched as hubs and heavily participate in multiple signaling pathways. Collectively, this comprehensive human kinome interactome map sheds light on anticancer drug resistance mechanisms and provides an innovative resource for rational kinase inhibitor design.

## INTRODUCTION

The human kinome has become one of the most important classes of drug targets in the pharmaceutical industry [1-3]. So far, more than 20 drugs targeting one or more kinases have been approved for clinical use in a variety of cancers, including lung, breast, melanoma, colorectal, pancreatic, and prostate cancers [1,4,5]. Moreover, as of 2012, more than 500 kinase inhibitors have been used as therapeutic drugs, approximately a third of which are undergoing clinical trials [4,6]. However, patients treated with those kinase inhibitors eventually develop resistance, and their prolong survivals are typically only a few months [5,7-12]. One reason for resistance is that kinases are extensively involved in complex biological mechanisms through adaptive crosstalk or feedback within cellular networks.

Most kinases are proteins, while others are lipids or small molecules. There are more than 600 putative kinase genes that account for ~3% of human protein-coding genes [13]. The kinases catalyze the reversible phosphorylation of ~500,000 phosphorylation sites in ~20,000-22,000 human proteins, playing critical roles in human cells as well as other eukaryotic cells. Furthermore, kinases are involved in various key cellular signaling pathways, including transcription, cancer cell metabolism, cell cycle progression, apoptosis, and differentiation [2,4,13]. It has been estimated that more than 400 human diseases are caused by kinase signaling pathway defects (http://www.kinasenet.ca/). So far, more than 80% of kinases have been investigated as drug targets for therapeutic development. However, a quantitative network measurement of functional relationships among drugs, kinases, and the human protein interactome at the kinome level remains largely unknown. Constructing a global human kinase phosphorylation network and the human kinome interactome resource is therefore essential to further explore the relationship among drug responses, network properties, and cellular functions, thereby accelerating rational kinase inhibitor design for cancer therapy.

In this study, we developed a systems biology-based framework to construct a global human kinome interactome map by integrating the kinase-substrate interaction network (KSIN), kinase-drug interaction network (KDIN), physical protein-protein interaction network (PPIN), and atomic resolution three-dimensional structural PPIN (3DPPIN). We systematically examined and compared the network topological and functional properties of several important gene or protein sets in this global human kinome interactome. These sets include kinase genes, Mendelian disease genes (MDGs), orphan disease-causing mutant genes (ODMGs), Cancer Gene Census (CGC) genes, essential genes, anticancer drug sensitivity genes, drug target proteins, and adverse drug reaction-associated proteins (ADRPs). We identified the conserved regulatory phosphorylation motifs (e.g., Ser/Thr-Pro) using a sequence logo analysis, which provides the evidence that the proline direction of kinases is a crucial mechanism in the conserved phosphorylation signaling pathways. We found that the distinct network centrality (e.g., hubs) of kinases creates a high risk for the evasion of single kinase target inhibition by feedback or crosstalk mechanisms. This notion is further supported by the systematic network and pathway analyses that anticancer drug resistance genes are significantly enriched as hubs and heavily participate in multiple cancer signaling pathways. Furthermore, we provided the statistical evidence that the typical anticancer target selection strategy, which uses network hubs as drug targets, might lead to a high risk for adverse drug reactions. Collectively, this study sheds light on kinase inhibitor resistance mechanisms and provides an innovative systems biology resource for rational kinase inhibitor design in individualized cancer therapy.

## RESULTS

**Figure 1:**
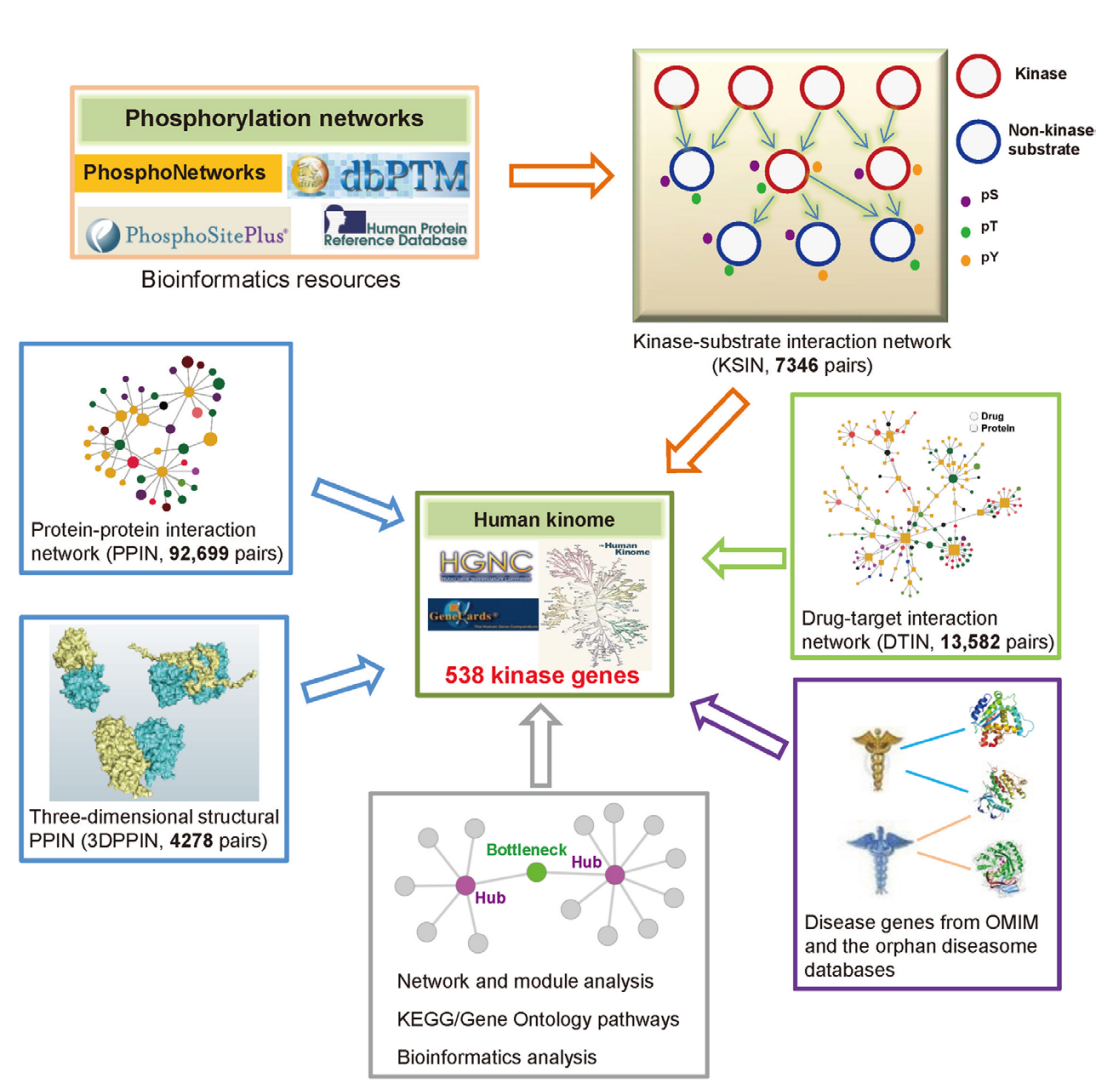
Diagram of systems biology-based framework for the human kinome interactome map building This human kinome interactome map across 538 kinase genes includes five components: (i) kinase-substrate interaction network, (ii) physical protein-protein interaction network (PPIN) and an atomic resolution three-dimensional structural PPIN, (iii) drug-target interaction network, (iv) disease gene annotations, and (v) network, pathways, and bioinformatics analyses.

**Figure 2:**
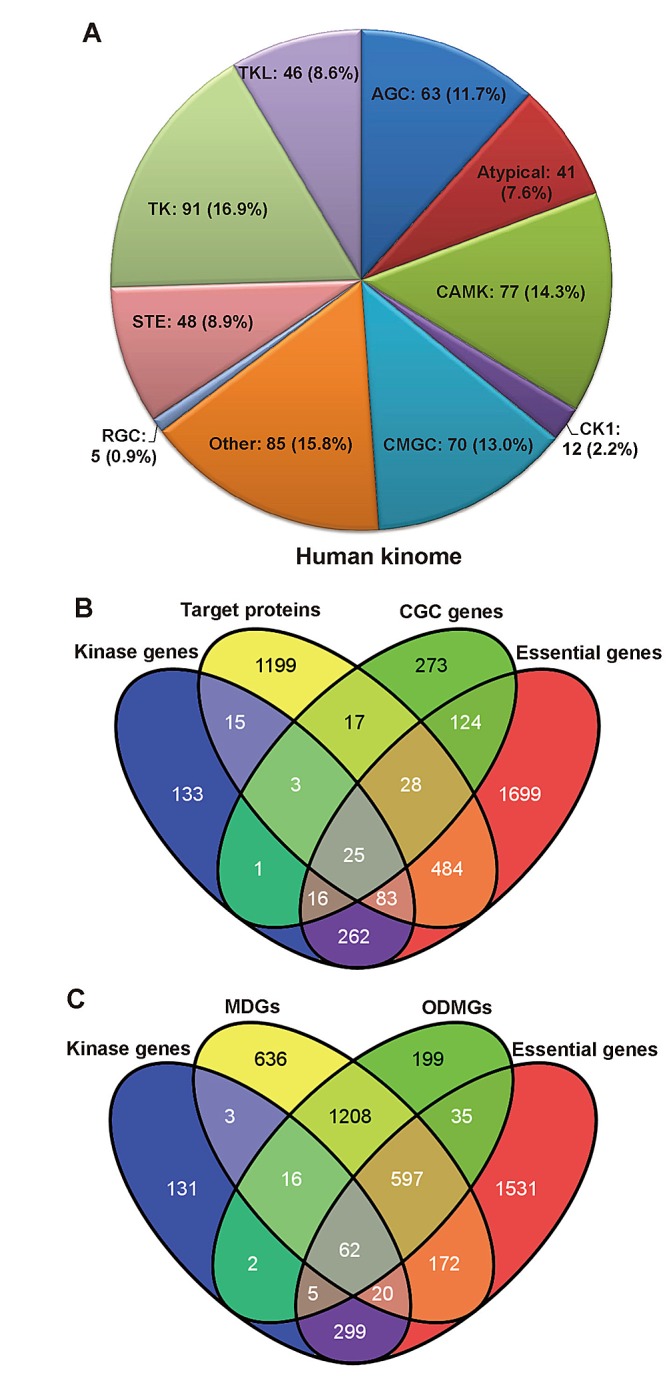
Functional annotations of the human kinome (A) Pie chart of 538 kinase genes grouped by 10 different kinase groups: tyrosine kinases (TK), tyrosine kinase-like kinases (TKL), casein kinases (CK1), PKA/PKG/PKC-family kinases (AGC), calcium/calmodulin-dependent kinases (CAMK), sterile homologue kinases (STE), CDK/MAPK/GSK3/CLK-family kinases (CMGC), receptor guanylate cyclases (RGC), atypical protein kinases (Atypical), and kinases that did not belong to any groups above (Other). (B) The Venn diagram of overlaps among 538 kinase genes, 1,855 drug target proteins, 487 Cancer Gene Census (CGC) genes, and 2,721 essential genes. (C) The Venn diagram of overlaps among 538 kinase genes, 2,714 Mendelian disease genes (MDGs), 2,123 orphan disease-causing mutant genes (ODMGs), and 2,721 essential genes.

We developed a systems biology-based framework (Figure 1) and used it to construct a global human kinome interactome map. The current version of the human kinome [13] includes 637 genes categorized into 10 groups: tyrosine kinases (TK), tyrosine kinase-like kinases (TKL), casein kinases (CK1), PKA/PKG/PKC-family kinases (AGC), calcium/calmodulin-dependent kinases (CAMK), sterile homologue kinases (STE), CDK/MAPK/GSK3/CLK-family kinases (CMGC), receptor guanylate cyclases (RGC), atypical protein kinases (Atypical), and kinases that did not belong to any group above (Other). After mapping them to the GeneCards and the National Center for Biotechnology Information (NCBI) [14] databases, 538 genes had official gene symbols and Entrez IDs (Supplementary Table S1). Figure 2A shows the distribution of these 538 kinase genes in 10 groups. Starting with these 538 kinase genes, we systematically constructed a global human kinome interactome map using the following data: a KSIN with 7346 pairs, a PPIN with 92,699 pairs, an atomic resolution 3DPPIN with 4278 pairs, and a drug-target interaction network with 13,582 pairs (Supplementary Table S1 and Figure 1). The collection of the human kinome and four networks is available at http://bioinfo.mc.vanderbilt.edu/kinomenetworkX/. Next, we systematically examined the topological features and functional relationships of these networks to better understand kinase inhibitor responses and molecular networks of the human kinome.

### Functional mapping of the human kinome

We compared the 538 kinase genes with each of the following gene sets: 487 CGC genes, 1,855 known drug target proteins (genes), 2,123 ODMGs, 2,714 MDGs, and 2,721 essential genes (Supplementary Table S1). Within the current human kinome, 422 kinase genes (78.4%) are found in at least one of these five gene sets, including 45 CGC genes, 126 drug target proteins (genes), 101 MDGs, 85 ODMGs and 386 essential genes (Figure 2). This observation indicated that kinase genes tended to be CGC genes more often as compared to MDGs (odds ratio=2.6, p=1.3×10^−6^, Fisher's exact test) or ODMGs (p=8.3×10^−6^). Among the 45 CGC kinase genes, 28 kinases have been approved by the United States Food and Drug Administration (FDA) for molecularly targeted cancer treatment. In order to further our understanding of the biological functions of the human kinome, we examined the cellular component features of 538 kinases using the ClueGO [15]. We found kinases tended to locate in the plasma membrane integral region (p=9.0×10^−9^, two-sided hypergeometric test), plasma membrane (p=6.1×10^−8^), cytoskeletal region (p=3.5×10^−5^), cytoskeleton (p=3.5×10^−4^), or cleavage furrow (p=4.5×10^−3^) (Supplementary Table S2). It is not surprising that kinases are enriched in membrane components, as the cell membrane is a key location for signal transduction and cell-cell communications.

### Kinase-substrate interaction network

We constructed a high-resolution KSIN using a systems biology-based framework in Figure 1. The current version of KSIN includes 7,346 experimentally validated or literature-curated kinase-substrate interaction (KSI) pairs connecting 379 kinases and 1,961 non-kinase substrate proteins (Supplementary Table S1). The details of kinase genes categorized by each kinase group are shown in Supplementary Figure S1. We further collected high-resolution *in vivo* phosphorylation sites from dbPTM3 [16] and PhosphositePlus [17], and used the data to annotate each protein kinase and its substrate protein. In total, we collected 173,460 non-redundant phosphorylation sites in 18,610 proteins (Supplementary Table S2). This collection included 94,693 phosphoserine (*p*S) sites (54.6%), 44,023 phosphothreonine (*p*T) sites (25.4%), and 34,744 phosphotyrosine (*p*Y) sites (20.0%) (Supplementary Figure S1C). Among these phosphorylation sites, 10,374 sites were found in a total of 490 kinases (91.1% of kinome), including 5,364 *p*S sites (51.7%), 2,581 *p*T sites (24.9%), and 2,429 *p*Y sites (23.4%) (Supplementary Figure S1D). Next, we compared the kinases and substrates in KSIN with their phosphoproteome sites. In total, 36,576 phosphorylation sites were found in 1,919 non-kinase substrate proteins in KSIN, including 21,184 *p*S sites (57.9%), 8,812 *p*T sites (24.1%), and 6,580 *p*Y sites (18.0%) (Supplementary Figure S1E).

### Topological characteristics of KSIN

**Figure 3:**
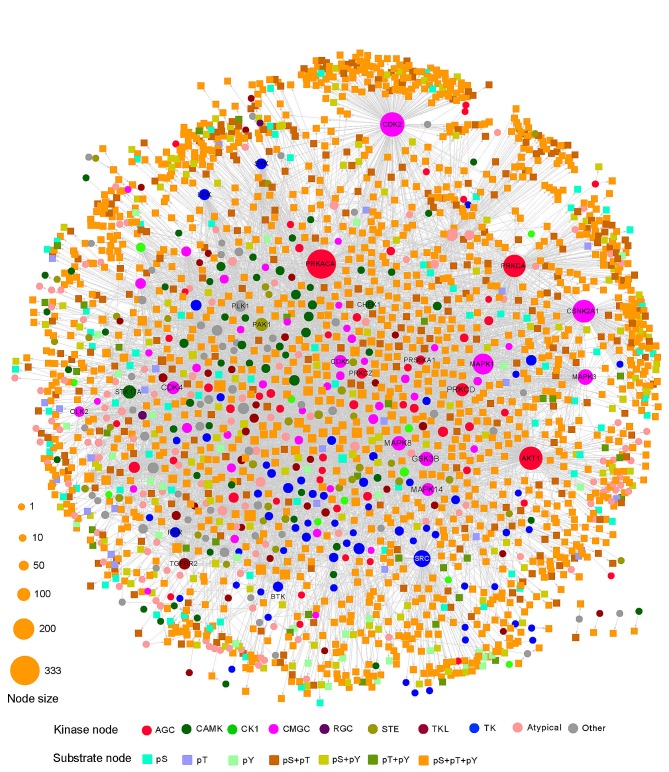
Kinase-substrate interaction network (KSIN) The size of each node reflects its degree of connectivity in KSIN. Abbreviations of kinase groups (circles) are provided in the Figure 2 legend. Non-kinase substrate nodes (squares) are color-coded according to their phosphorylation sites, including phosphoserine (*pS*), phosphothreonine (*pT*), and phosphotyrosine (*pY*).

We visualized KSIN in Cytoscape and examined its network topological characteristics in Figure 3. In this network, 379 kinases were denoted by circles, and 1,961 non-kinase substrate proteins were denoted by squares. A straightforward exploration of the network revealed several major hubs, including PRKACA (connectivity=333), CDK2 (255), AKT1 (234), CSNK2A1 (227), PRKAC (223), MAPK1 (194), SRC (163), MAPK3 (141), MAPK3 (124), and GSK3B (123), all of which were involved in multiple substrate protein phosphorylation reactions. An examination of the connectivity distribution of KSIN showed that it follows a power-law distribution (y=axb, a=380.1, b=-1.3), with an average connectivity of 6.3 and an average shortest path of 3.5 (Supplementary Figure S1F).

### Modularity characteristics

Network modules, also known as network communities, represent groups of interconnected nodes that typically have similar biological functions. We used CFinder [18] to identify functional modules of KSIN. A total of 21 functional modules (Supplementary Table S2) were generated by CFinder (*k*-clique, *k*=4). The giant module included 733 KSI pairs connecting 110 kinases and 140 substrate proteins. Modules were further analyzed for overlaps and correlations using the ModuLand algorithm [19,20]. In total, we identified 89 overlapped modules (Supplementary Figure S2A). The correlation of 89 modules is shown in Supplementary Figure S2B.

### Substrate specificity of the human kinome

**Figure 4:**
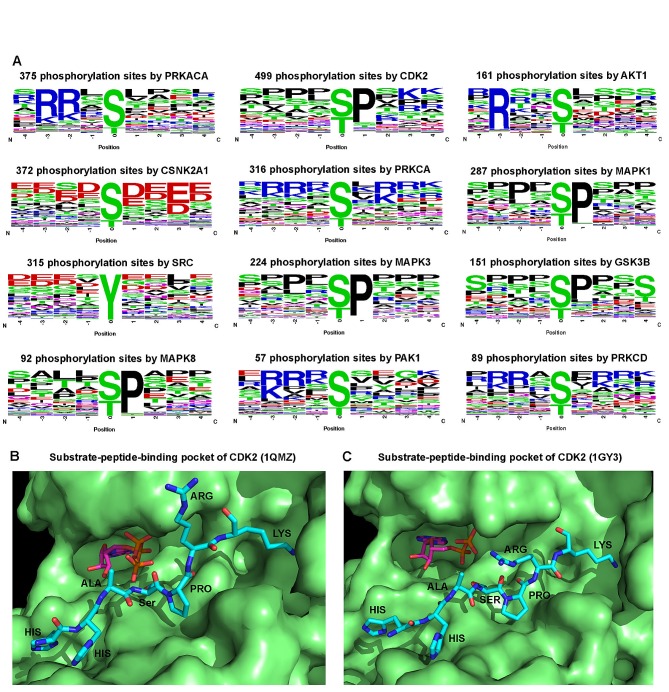
Sequence motif analysis of kinase phosphorylation sites (A) Logo analysis of target phosphorylation site sequence motifs (four amino acids before and after the phosphorylation residues) for 12 kinases that have the strongest connectivity in kinase-substrate interaction network. The amino acids are labeled according to their chemical properties: green for polar amino acids (G, S, T, Y, C, Q, N), blue for basic amino acids (K, R, H), red for acidic amino acids (D, E), and black for hydrophobic amino acids (A, V, L, I, P, W, F, M). (B) A substrate peptide binding pocket of CDK2 (PDB ID: 1QMZ). (C) Another substrate peptide binding pocket of CDK2 (PDB ID: 1GY3). B and C were prepared using the software PyMOL (http://www.pymol.org/).

We examined sequence motifs of phosphorylation site using a sequence logo analysis tool [21]. Supplementary Table S2 shows the results for the targeted phosphorylation sites of the top 12 kinases that have the highest connectivity in KSIN. We found kinases recognized distinct sequence motifs (Figure 4A). For example, a serine or threonine residue preceding a proline (Ser/Thr-Pro) is a major regulatory phosphorylation motif that plays crucial functions in a diverse array of cellular processes.[22] Figure 4A showed several important functional hubs, including CDK2, MAPK1, MAPK3, and MAPK8 that harbored the conserved Ser/Thr-Pro motif. Similarly, glycogen synthase kinase-2 (GSK3B) is more likely to recognize and phosphorylate the first serine in the conserved motif Ser-X-X-X-Ser-Pro [23]. The Ser/Thr-Pro-directed kinases play crucial roles in cell cycle, transcription, and diverse signaling transduction pathways as well as in Alzheimer's disease and various cancers [24]. For CSNK2A1, its +1 position has an Asp/Glu (Figure 4A), confirming that CSNK2A1 is a Ser-Asp/Glu-directed kinase [25]. The -3 and -2 positions of PRKACA, AKT1, PAK1, PRKCA, and PRKCD form conserved phosphorylation consensus motifs, such as Arg-Arg-X-Ser/Thr and Arg/Lys-X-X-Ser/Thr [26]. Moreover, distinct regulatory phosphorylation motifs were verified by kinase-substrate co-crystal structures. The pocket of phospho-CDK2-cyclinA3-peptide complex [27,28] tended to accommodate proline at the +1 position (Figure 4B,C). However, it should be noted that analyses here are limited due to the incompleteness and inaccuracy of existing data.

### Kinase-drug interaction network

**Figure 5:**
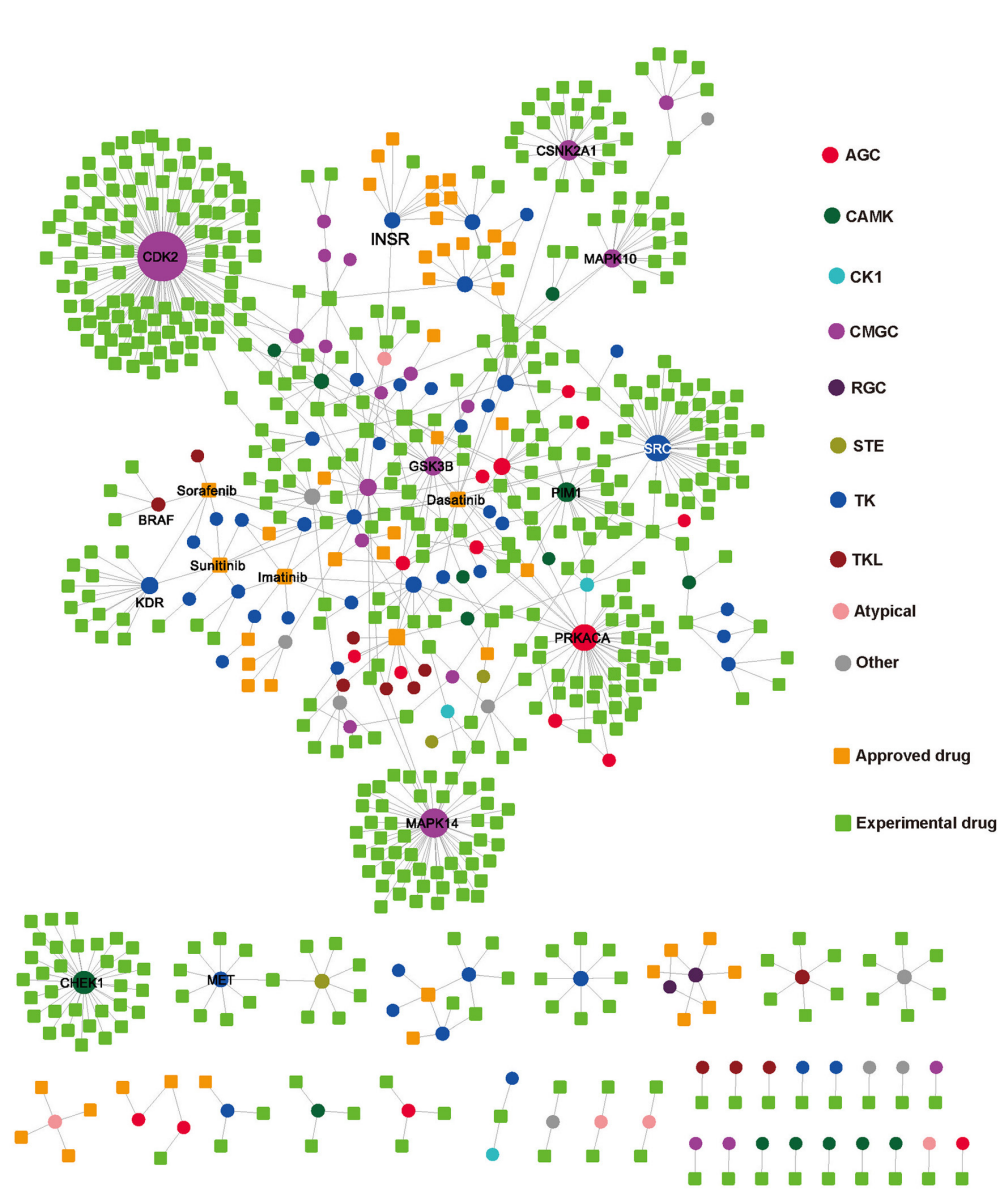
Kinase-drug interaction network In this network, a drug node (square) and a target kinase node (circle) are connected to each other by a grey edge if the target is annotated as a known interaction with the drug. The size of each node reflects its degree of connectivity. Drug nodes (circles) are green (experimental drugs) or gold (FDA approved drugs). Kinase nodes (circles) are color-coded according to the kinase groups (see Figure 2 legend).

We searched drugs that target any of the 538 kinases from DrugBank [6] and Therapeutics Target Database (TTD) [29] and found a total of 567 drugs targeting 126 kinases (Supplementary Table S2, as of April 30, 2013). Then, we used these drugs and their target kinases to build a bipartite graph of the kinase-drug interaction network (KDIN) in Figure 5. The bipartite graph analysis of KDIN could provide a useful survey of the current status of kinase inhibitor discovery and clinical applications. In KDIN, a drug (square) and a kinase (circle) are linked if the kinase is a known target of the drug (Figure 5). The average connectivity (4.6) of 11 FDA approved small molecularly targeted kinase inhibitors is significantly stronger than that of 527 experimental drugs (1.2, p=1.6×10^−8^, Wilcoxon test). The bipartite graph analyses showed that most FDA-approved kinase inhibitors often target the cancer kinome through polypharmacology. For instance, dasatinib is an oral dual ABL1 and SRC family tyrosine kinase inhibitor for chronic myelogenous leukemia treatment. As shown in Figure 5, dasatinib targets 9 protein kinases, including ABL1, ABL2, EPHA2, KIT, PDEGFRB, FYN, SRC, YES1, and LCK. Furthermore, a kinase may be targeted by multiple drugs. For example, CDK2 binds 142 experimental drugs in DrugBank and TTD. Since most tumors could evade the inhibition of any single kinase [5], development of a polypharmacological inhibitor would be a promising strategy to improve clinical benefits for cancer therapy [5,30,31]. The two-dimensional chemical structures, detailed annotation data, and FDA-approved clinical usages of 11 small molecular kinase inhibitors are provided in Supplementary Figure S3.

### Topological properties of the human kinome interactome

Do hubs in the protein interactome tend to be drug targets? In KSIN, 468 proteins within the top 20% of connectivity were selected as hubs. After manually mapping, we found 116 target kinases in KSIN, of which 90 were hubs (p=3.6×10^−43^, Fisher's exact test, Table 1). In PPIN, 2,602 proteins within the top 20% of connectivity were selected as hubs. After manual mapping, we found 125 target kinases, among which 90 target kinases were hubs (p=2.5×10^−35^, Table 1). This target kinase enrichment in hubs was observed in 3DPPIN as well (p=1.5×10^−22^).

**Table 1:** The network topological analysis for five gene sets in human protein interactome

Network	Gene sets	Number of hubs	Number of non-hubs	Odds ratio	p-value
KSIN	Kinome	277	152	16.4	1.2×10^−119^
	Drug target kinases	90	26	16.9	3.6×10^−43^
	Drug target proteins	121	288	1.9	2.8×10^−7^
	ADRPs	54	105	2.2	2.0×10^−5^
	Drug sensitivity genes	40	84	2.0	1.1×10^−3^
PPIN	Kinome	209	253	3.4	5.9×10^−34^
	Drug target kinases	90	35	10.2	2.5×10^−35^
	Drug target proteins	438	1112	1.6	1.3×10^−14^
	ADRPs	122	319	1.5	3.1×10^−4^
	Drug sensitivity genes	137	245	2.2	3.0×10^−12^
3DPPIN	Kinome	117	154	3.0	2.1×10^−15^
	Drug target kinases	68	33	7.8	1.5×10^−22^
	Drug target proteins	162	293	2.2	3.2×10^−12^
	ADRPs	47	75	2.2	5.4×10^−5^
	Drug sensitivity genes	58	75	2.8	3.2×10^−8^

KSIN: kinase-substrate interaction network (468 hubs versus 1,872 non-hubs). PPIN: protein-protein interaction network (2,602 hubs versus 10,041 non-hubs). 3DPPIN: three-dimensional structural PPIN (591 hubs versus 2,018 non-hubs). ADRPs: adverse drug reaction-associated proteins. The p-value was calculated using Fisher's exact test.

Furthermore, we compiled 13,582 drug-target interactions to construct a complementary drug target protein network (DTPN). In DTPN, nodes are target proteins, and two target proteins are connected if they are both targeted by at least one common FDA-approved or experimental drug [32]. This DTPN included 28,989 pairs connecting 1,811 target proteins. Statistical analysis showed that DTPN target proteins were more likely KSIN hubs (p=2.8×10^−7^, Table 1). There were 1,550 target proteins shared by DTPN and PPIN. Within these proteins, 438 (28.3%) were hubs, indicating a significant enrichment of DTPN target proteins in PPIN hubs (p=1.3×10^−14^). The same enrichment was found when 3DPPIN was compared to DTPN (p=3.2×10^−12^). Collectively, drug target proteins (e.g., target kinases) were more likely to be hubs in KSIN, PPIN, and 3DPPIN.

The emerging use of network hubs as drug targets has the following rationale [33]. Perturbation of hubs by a drug would create cascading effects, leading to functional changes in a major segment of the network. In contrast, peripheral nodes (non-hubs) that are blocked by a molecule would likely have only limited effects. However, our analyses below revealed that selecting network hubs as drug targets lead to a high risk of adverse drug reactions. We compiled 527 ADRPs that are involved in adverse drug reactions. When 527 ADRPs were manually matched to PPIN, 441 proteins were found, among which 122 proteins were hubs in PPIN (p=3.1×10^−4^, Table 1). In addition, the ADRPs were significantly enriched as network hubs in KSIN (p=2.0×10^−5^) and 3DPPIN (p=5.4×10^−5^). Therefore, there is a high risk for adverse drug reactions when using the hubs in the human protein interactome as drug targets.

To further investigate whether targeting a signaling pathway is more effective, we used the ClueGO [15] to identify KEGG pathways enriched with the 126 target kinases. Three important signaling pathways were identified: MAPK signaling pathway (including EGFR, BRAF, PDGFR, MAPK1, TGFBR1, and RAF1, p=2.3×10^−15^, two-sided hypergeometric test, Supplementary Table S3), VEGF signaling pathway (including** SRC, PRKCA, MAPK, and KDR, p=1.3×10^−12^), and mTOR signaling pathway (including BRAF, AKT1, RPS6KA1, and mTOR, p=1.7×10^−7^).

### Do kinases tend to be hubs in the human protein interactome?

We manually matched 538 kinases to PPIN and then constructed a kinase-protein interaction subnetwork. This subnetwork included 14,238 pairs connecting 462 kinases and 4,414 non-kinase proteins (Supplementary Table S4). Among the 462 kinases, 209 were hubs in PPIN, indicating a significant enrichment of kinases in PPIN hubs (p=5.9×10^−34^, Table 1). The average connectivity of the 462 kinases was 33.2, which is significantly stronger than that of the 12,181 non-kinases in PPIN (14.0, p<2.2×10^−16^, Wilcoxon test, Supplementary Table S4). We further matched 538 kinases in 3DPPIN and found 271 kinases, including 117 hubs (p=2.1×10^−15^, Table 1). The average connectivity (6.0) of the 271 kinases is significantly stronger than that of the 2,338 non-kinases in 3DPPIN (3.0, p<2.2×10^−16^). Collectively, kinases are significantly enriched as network hubs in the protein interactome.

### Do hubs in KSIN tend to be hubs/bottlenecks in the protein interactome?

We manually matched 2,340 proteins in KSIN to PPIN. A total of 2,213 proteins (including 361 kinases and 1,852 non-kinase substrates) were found, among which 1,119 proteins (including 194 kinases and 925 non-kinases) were hubs in PPIN (p=3.6×10^−275^, Supplementary Table S4). In addition, 965 proteins (including 169 kinases and 796 non-kinases) were bottlenecks in PPIN (p=1.1×10^−166^). The average connectivity (9.8) of the 965 bottleneck proteins is significantly stronger than that of the 1,248 non-bottleneck proteins in KSIN (3.8, p<2.2×10^−16^). These findings revealed that proteins in KSIN tended to be bottlenecks in PPIN.

### Network topology of anticancer drug response-associated genes

A systematic identification of anticancer drug response markers in cancer cells is highly promising for individualized cancer therapy [34]. In this study, we sought to determine the network topology of drug resistance genes in the protein interactome. We compiled 458 genes that are involved in sensitivity or resistance to 130 anticancer drugs from a previous work [35]. Among the 458 drug resistance genes, 82 were CGC genes and 144 were essential genes (Supplementary Figure S4A). We found 124 among the 458 drug resistance proteins (genes) in KSIN, 40 of them were hubs, suggesting a significant enrichment of drug resistance proteins in KSIN hubs (p=1.1×10^−3^, Fisher's test, Table 1). The average connectivity (10.5) of the 124 drug resistance proteins was significantly stronger than that of the 2,216 remaining proteins in KSIN (6.1, p=2.6×10^−4^, Wilcoxon test, Supplementary Table S5). Furthermore, we found a significant enrichment of anticancer drug resistance proteins in PPIN hubs (137 hubs, p=3.0×10^−12^) and 3DPPIN hubs (58 hubs, p=3.2×10^−8^).

**Figure 6:**
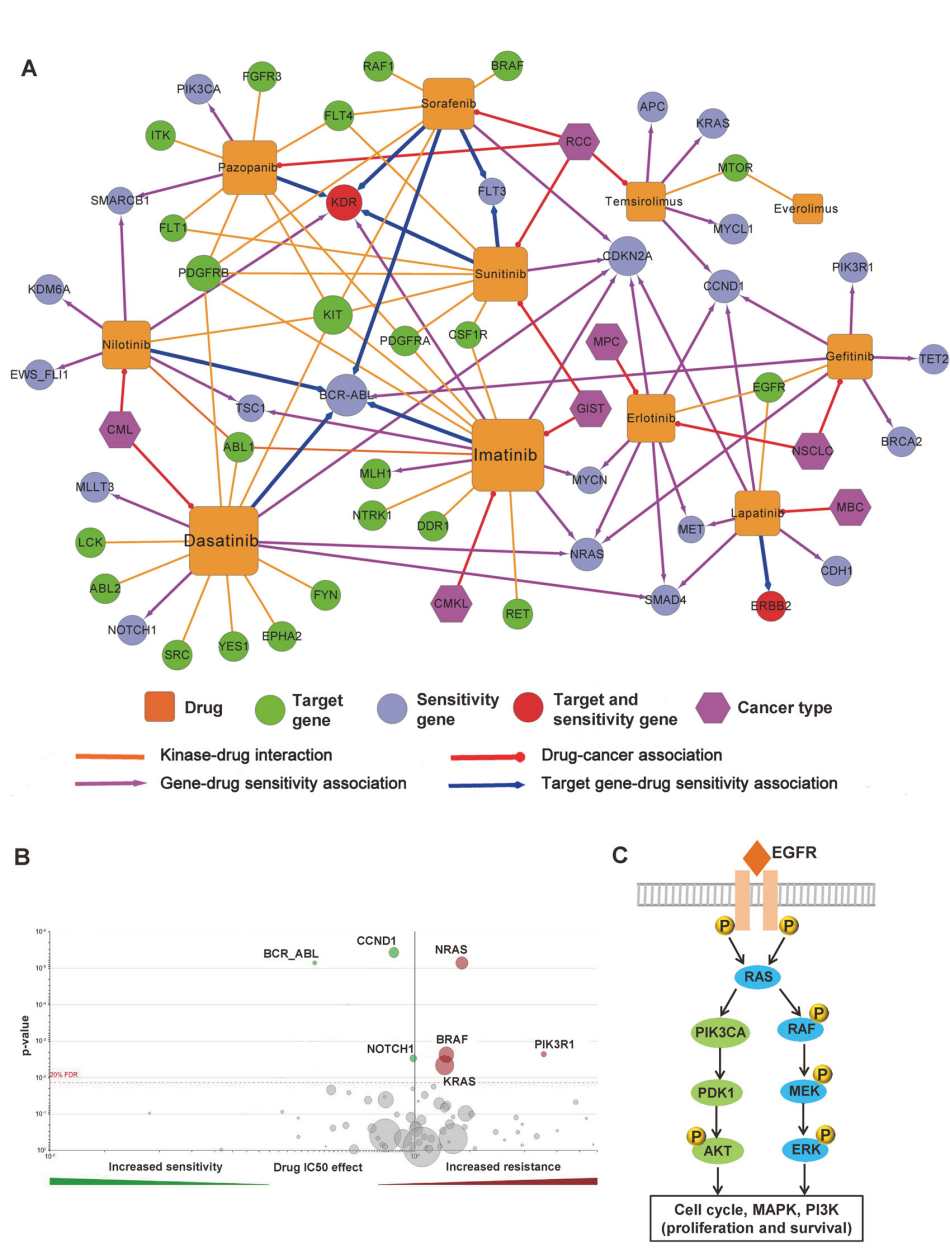
Network analysis of kinase inhibitor response (A) Drug sensitivity network of 11 molecularly targeted kinase inhibitors (Supplementary Figure S5). This network includes four types of edges: kinase-drug interaction (gold solid line), drug-cancer association (red solid line), gene-drug sensitivity associations (purple solid line with arrow), and target gene-drug sensitivity associations (blue solid line with arrow). Color codes of nodes: drug (gold square), target gene or target protein (green circle), drug sensitivity genes (cyan-blue circle), drug target and sensitivity gene (red circle), and cancer (purple hexagon). (B) Volcano plot of sensitivity response to Gefitinib, an epidermal growth factor receptor (EGFR) inhibitor. The calculation of a p-value for each drug-gene association was described in a previous work [35]. The data was from the Genomics of Drug Sensitivity (http://www.cancerrxgene.org). (C) The simplified EGFR signaling pathways involving Gefitinib sensitivity through the RAS/MEK/ERK and PI3K/PDK1/AKT pathways.

Next, we constructed a drug resistance network to investigate the detailed molecular mechanisms of drug responses to specific FDA-approved small molecular kinase inhibitors (Supplementary Figure S3). The calculation of a p-value for each drug-gene association was described in a previous work [35]. Three kinase inhibitors are approved for renal cell carcinoma treatment by the FDA, including sunitinib, sorafenib, and pazopanib (Figure 6A). The kinase inert domain receptor (KDR) is involved in resistance or sensitivity to sunitinib (p=1.9×10^−4^), sorafenib (p=1.0×10^−3^), and pazopanib (p=1.0×10^−3^) (Supplementary Table S5). The cyclin-dependent kinase inhibitor 2A gene (*CDKN2A*), which encodes the CDK inhibitory protein p16, was reported to be significantly associated with sensitivity to five kinase inhibitors (Supplementary Table S5): dasatinib (p=1.3×10^−13^), erlotinib (p=4.1×10-^8^), imatinib (p=8.2×10^−5^), sunitinib (p=5.0×10^−3^), sorafenib (p=6.0×10^−3^), and lapatinib (p=7.0×10^−3^). Gefitinib, a classical EGFR tyrosine kinase inhibitor, is approved to treat advanced or metastatic non-small cell lung cancer. Recent work showed genes *NRAS*, *BRAF*, *KRAS*, and *PIK3R1* are involved in gefitinib resistance (Figure 6B) [36]. In Figure 6C, most gefitinib resistance genes are located on the EGFR signaling pathway through the RAS/MEK/ERK or PI3K/PDK1/AKT downstream pathways [34]. Collectively, selecting a network hub as the drug target in the protein interactome might create a high anticancer drug resistance risk.

## DISCUSSION

Resistance to chemotherapy and molecularly targeted kinase inhibitor therapeutics is a major obstacle facing current cancer research [37]. Crosstalk and feedback that are poorly understood in most cellular networks are main contributors to resistance. Systems biology-based modeling on the human kinome level might provide a powerful network perspective and innovative tools to address this challenge. In this study, we systematically investigated the relationship between kinase inhibitors and the gene products of the human kinome in the broader context of the human kinase phosphorylation network and the protein interactome. We focused on addressing two questions. First, do kinases occupy a distinct network topology in the human interactome? Second, from a systems biology perspective, why do most tumors escape from any single kinase inhibition? We found kinases significantly tended to be central hubs rather than peripheral nodes in the protein interactome. The distinct network centrality of kinases creates a high risk for the evasion of single kinase target inhibition through adaptive feedback or crosstalk within dynamic signaling networks. Moreover, this hypothesis is further supported by the systematic network and pathway analyses that anticancer drug sensitivity proteins are significantly enriched as hubs in the protein interactome. We further revealed that the typical anticancer drug target selection strategy that uses hubs as drug targets, might lead to a high risk for adverse drug reactions. In summary, these findings provide systems view of the human kinase interactions and kinase inhibitor resistance mechanisms.

Phosphorylation-mediated signaling networks play crucial roles in cellular physiology. Recent protein microarray experiments have provided high-throughput data and facilitated the analyses of protein phosphorylation networks [38]. Here, we constructed a global and high-resolution kinase phosphorylation network using an integrative computational framework. We identified conserved regulatory phosphorylation motifs, e.g., Ser-X-X-X-Ser-Pro, using a sequence logo analysis. These conserved regulatory phosphorylation motifs were verified by the kinase-substrate co-crystal structures. Thus, we provided the evidence that the proline direction of kinases is a common mechanism for the conserved phosphorylation signaling pathways. The data size used in this study is reasonable and includes the data we were able to collect to date. However, some important properties might not be captured among this data due to knowledgebase incompleteness as well as the standard static networks that are prevalent in this field. Biological systems, e.g., the phosphorylation network, are highly dynamic profiles that continuously respond to a host of physical or physiological environments. So far, the completeness and accuracy of the human interactome is still a major obstacle. For example, the perturbation dynamics of signaling networks have been extensively investigated, including ~10,000 phosphorylation reactions in yeast cells [39]. The size of the whole human interactome was estimated to have ~650,000 interactions [40]. Although two large networks were constructed in this study, we still have a long way to decipher the complexity of the human kinome interactome. Advances in experimental measurement technologies and computational methods would enable large-scale screenings to fill in much of our missing knowledge in the future.

Here, we systematically examined the kinase-drug interaction network using a bipartite graph analysis. We found target kinases are significantly enriched as central hubs in the protein interactome. An inhibition or blocking of hub nodes may lead to cascading effects compromising the function of a major segment of the signaling networks [33]. The development of high efficacy kinase inhibitors that target hubs of the signaling networks is a typical strategy for cancer drug discovery [41]. However, network centrality of target kinases might create a high risk of drug resistance, as hub proteins easily provide the adaptive crosstalk or feedback within cellular networks. In addition, we found the current selection of hubs as drug targets might create a high risk for the adverse drug reactions. Many promising drug candidates fail in the last clinical trial phases due to a poor understanding of the signaling pathways or drug-target interactions that are involved in the mechanisms-of-action [42]. To overcome this challenge, we urge researchers to expand the knowledge of systems pharmacology through the construction of network models [5,43]. Here, we found most of the successful kinase inhibitors primarily target the cancer kinome through polypharmacology. The polypharmacology of kinase inhibitors would improve clinical efficiency by inhibiting multiple kinases in the signaling networks [5,33]. Thus, network-based modeling potentially opens a new avenue for rational kinase inhibitor discovery, e.g., “Allo-network drugs” development [33,34]. The human kinome interactome we constructed in this study, named Kinome NetworkX, is available at http://bioinfo.mc.vanderbilt.edu/kinomenetworkX/. This comprehensive data source would serve as a useful resource for the research community. Collectively, the global human kinome interactome map provide a systems biology perspective for the human kinome, and this map is a useful resource for rational kinase inhibitor design in individualized cancer therapy.

## MATERIAL AND METHODS

### Construction of the human interactome

### Kinase-substrate interaction network (KSIN)

In KSIN, a node denotes a kinase or its substrate protein, and an edge denotes a phosphorylation reaction between a kinase and its substrate protein. We collected high-resolution KSI pairs from four databases: Phospho. ELM [44], Human Protein Resource Database (HPRD) [45], PhosphoNetworks [38,46], and PhosphoSitePlus [17]. All genes were mapped to their Entrez ID based on the NCBI [14] as well as their official gene symbols based on GeneCards (http://www.genecards.org/). Duplicated KSI pairs and self-loops were removed. As a result, we compiled 7,346 unique KSI pairs connecting 379 kinases and 1,961 non-kinase substrate proteins. In addition, we collected human phosphorylation site information from the PhosphoSitePlus [17] and dbPTM3 [16] databases. In total, we obtained 173,460 non-redundant phosphorylation sites in 18,610 proteins.

### Protein-protein interaction network (PPIN)

We downloaded human protein-protein interaction (PPI) pairs from the Protein Interaction Network Analysis (PINA) platform. PINA (v2.0, May 1, 2013) is a comprehensive PPI database that integrates six large-scale, manually curated public databases: IntAct, MINT, BioGRID, DIP, HPRD, and MIPS MPact [47]. All protein-coding genes were mapped to the NCBI database. Genes without an Entrez ID, duplicated PPI pairs, and self-loops were excluded. In total, we obtained 92,699 unique PPI pairs connecting 12,643 proteins.

### Three-dimensional structural protein-protein interaction network (3DPPIN)

We downloaded three-dimensional structural PPI (3DPPI) pairs from the Instruct database [48]. The original Instruct database contained 6,534 human 3DPPI pairs. After excluding genes without Entrez IDs and 2,293 self-loops, we collected 4,278 3DPPI pairs connecting 2,609 proteins.

Drug-target interaction network We collected the drug-target interactions from two famous drug pharmacological databases: DrugBank [6] and TTD [29]. In total, we collected 13,582 drug-target interaction pairs connecting 2,716 target proteins and 3,779 FDA-approved and experimental drugs.

### Gene set categories

### Mendelian disease genes (MDGs)

We downloaded 2,714 MDGs from the Online Mendelian Inheritance in Man (OMIM) database (December 2012) [49]. The OMIM contained 4,132 gene-disease association pairs connecting 2,716 disease genes in 3,294 Mendelian diseases or disorders (December 2012).

### Orphan disease-causing mutant genes (ODMGs)

We collected 2,123 ODMGs from a previously published work [50]. According to the United States Rare Disease Act of 2002, an orphan disease is defined as a rare disease that affects fewer than 200,000 inhabitants, which is equivalent to approximately 6.5 patients per 10,000 inhabitants [51].

### Cancer Gene Census (CGC) genes

We collected 487 cancer genes from the Cancer Gene Census (CGC, http://cancer.sanger.ac.uk/cancergenome/projects/census/). CGC genes are well-curated and have been widely used as a reference cancer gene set in many cancer-related projects [52,53].

### Essential genes

We downloaded 2,721 essential genes from the Online GEne Essentiality (OGEE) database [54]. Essential genes, whose knockouts result in cell inviability or embryonic lethality, are a crucial component to the study of biological systems robustness and effective drug target identification [54].

### Adverse drug reaction-associated proteins (ADRPs)

We compiled 546 ADRPs from a previously published work [55]. ADRPs are proteins that mediate adverse drug reactions or toxicity by binding to drugs or their reactive metabolites. Duplicated proteins and genes without Entrez IDs were excluded, resulting in 527 ADRPs.

### Anticancer drug response-associated genes

We collected 458 genes that were involved in the sensitivity or resistance to 130 anticancer drugs from a previous work [35]. In this study, Mathew et al. systematically identified drug-sensitivity biomarkers (genes) on 639 human tumor cell lines, which provided a useful resource to probe drug sensitivity genes.

### Measurement of network topology

We calculated connectivity (degree) and betweenness centrality values using the Cytoscape (v3.0) [56]. We defined “hubs” as those nodes that were ranked at the top 20% of the connectivity distribution and “bottleneck” as those nodes that were ranked at the top 20% of the betweenness centrality value distribution [50,57]. We identified network modules and communities using CFinder [18] (*k*-clique, *k*=4) and the ModuLand algorithm [19,20]. CFinder was used to locate and visualize overlapping, densely interconnected groups of nodes in undirected graphs [18]. The ModuLand algorithm was used to identify hierarchical layers of overlapping network modules and community centrality [19,20].

### Functional enrichment analysis

We used ClueGo [15], a user-friendly Cytoscape plug-in, for the enrichment analysis of genes in Gene Ontology cellular components or KEGG canonical pathways. A two-sided hypergeometric test was performed to estimate statistical significance.

### Statistical analysis and network visualization

All statistical tests (e.g., Fisher's exact test and Wilcoxon's test) were performed on the R platform (v3.01, http://www.r-project.org/). All network visualization and related network topological parameters were presented using Cytoscape (v3.0) [56].

## SUPPLEMENTARY INFORMATION AND MATERIAL










