# Supplementary material for: Quantitative network mapping of the human kinome interactome reveals new clues for rational kinase inhibitor discovery and individualized cancer therapy
Source: Oncotarget. 2014 May 18;5(11):3697–710. doi: 10.18632/oncotarget.1984 (PMC4116514; doi:10.18632/oncotarget.1984)
Supplement: Supplementary file 5 [file oncotarget-05-3697-s005.pdf]

Quantitative network mapping of the human kinome interactome reveals new clues for rational kinase inhibitor discovery and individualized cancer therapy

Supplementary Material

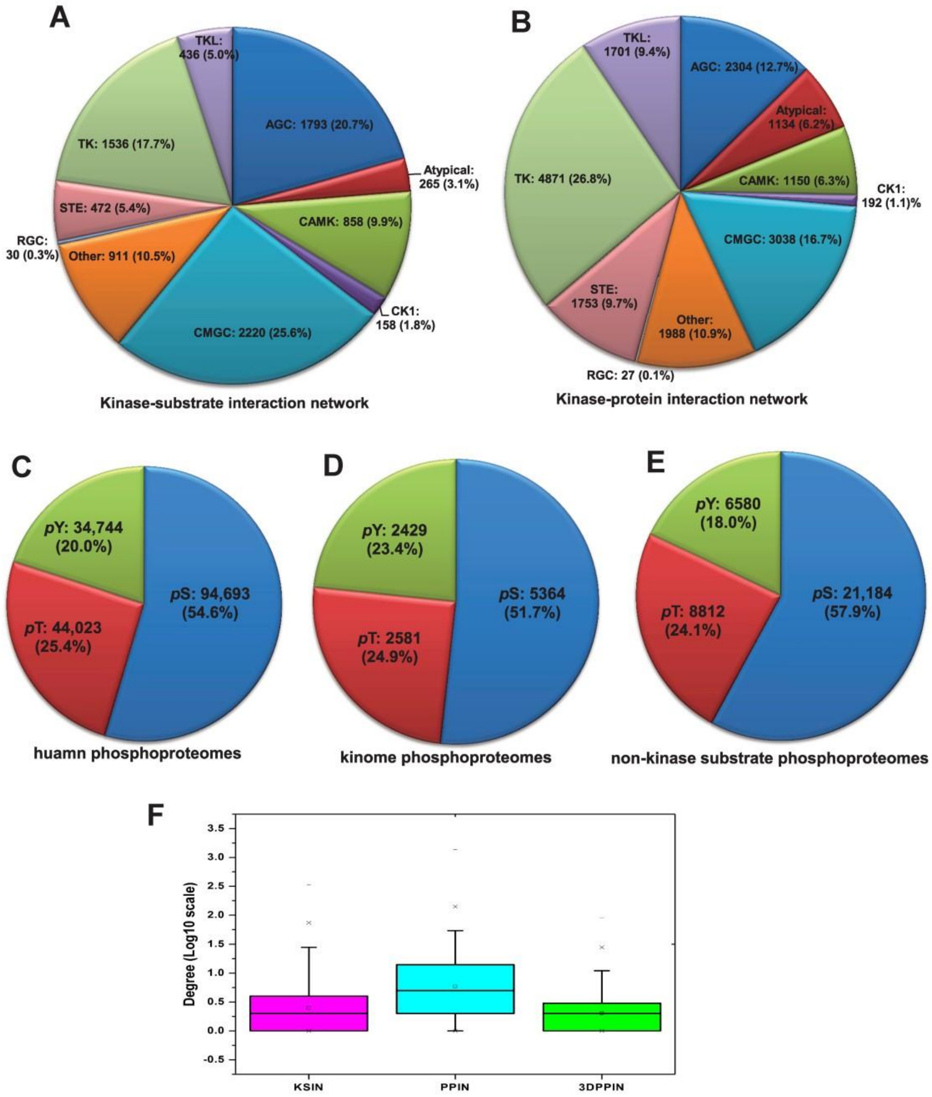

Figure S1: The distribution of kinase-substrate interactions, kinase-protein interactions, phosphorylation sites, and potein connectivity in three networks. A and **B**: The distribution of kinase-substrate interaction and kinase-protein interaction subnetwork grouped by 10 kinase groups. **C**, **D** and **E**: The distribution of phosphorylation sites (Threonine, Tyrosine and Serine) for the human phosphoproteomes (**C**, 18,610 proteins), kinome (**D**, 490 kinases) and non-kinase substrate proteins (**E**, 1,919 proteins). **F**: The blot plots of protein connectivity distribution in three networks: kinase-substrate interaction network (KSIN), protein-protein interaction network (PPIN), and three-dimensional structural protein-protein interaction network (3DPPIN). The detailed data are provided in Supplementary Table S1 and S2.

A

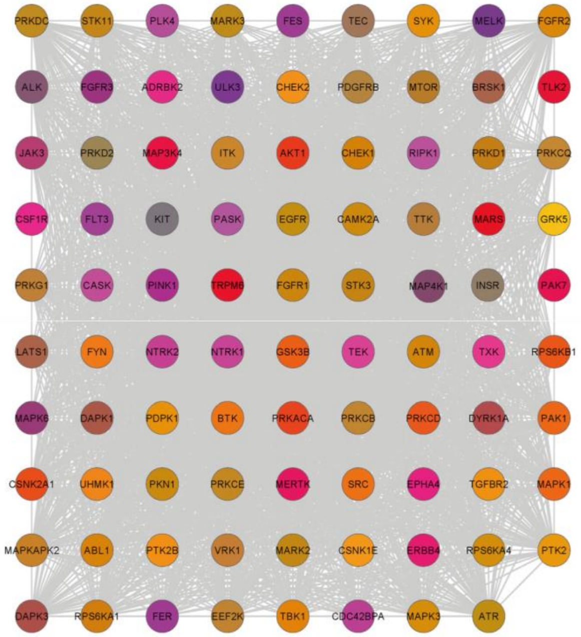
PRKDC
STK11
PLK4
MARK3
FES
TEC
SYK
MELK
FGFR2
ALK
FGFR3
ADRBK2
ULK3
CHEK2
PDGFRB
MTOR
BRSK1
TLK2
JAK3
PRKD2
MAP3K4
ITK
AKT1
CHEK1
RIPK1
PRKD1
PRKCQ
CSF1R
FLT3
KIT
PASK
EGFR
CAMK2A
TTK
MARS
GRK5
PRKG1
CASK
PINK1
TRPM6
FGFR1
STK3
MAP4K1
INSR
PAK7
LATS1
FYN
NTRK2
NTRK1
GSK3B
TEK
ATM
TXK
RPS6KB1
MAPK6
DAPK1
PDPK1
BTK
PRKACA
PRKCB
PRKCD
DYRK1A
PAK1
CSNK2A1
UHMK1
PKN1
PRKCE
MERTK
SRC
EPHA4
TGFBR2
MAPK1
MAPKAPK2
ABL1
PTK2B
VRK1
MARK2
CSNK1E
ERBB4
RPS6KA4
PTK2
DAPK3
RPS6KA1
FER
EEF2K
TBK1
CDC42BPA
MAPK3
ATR

B

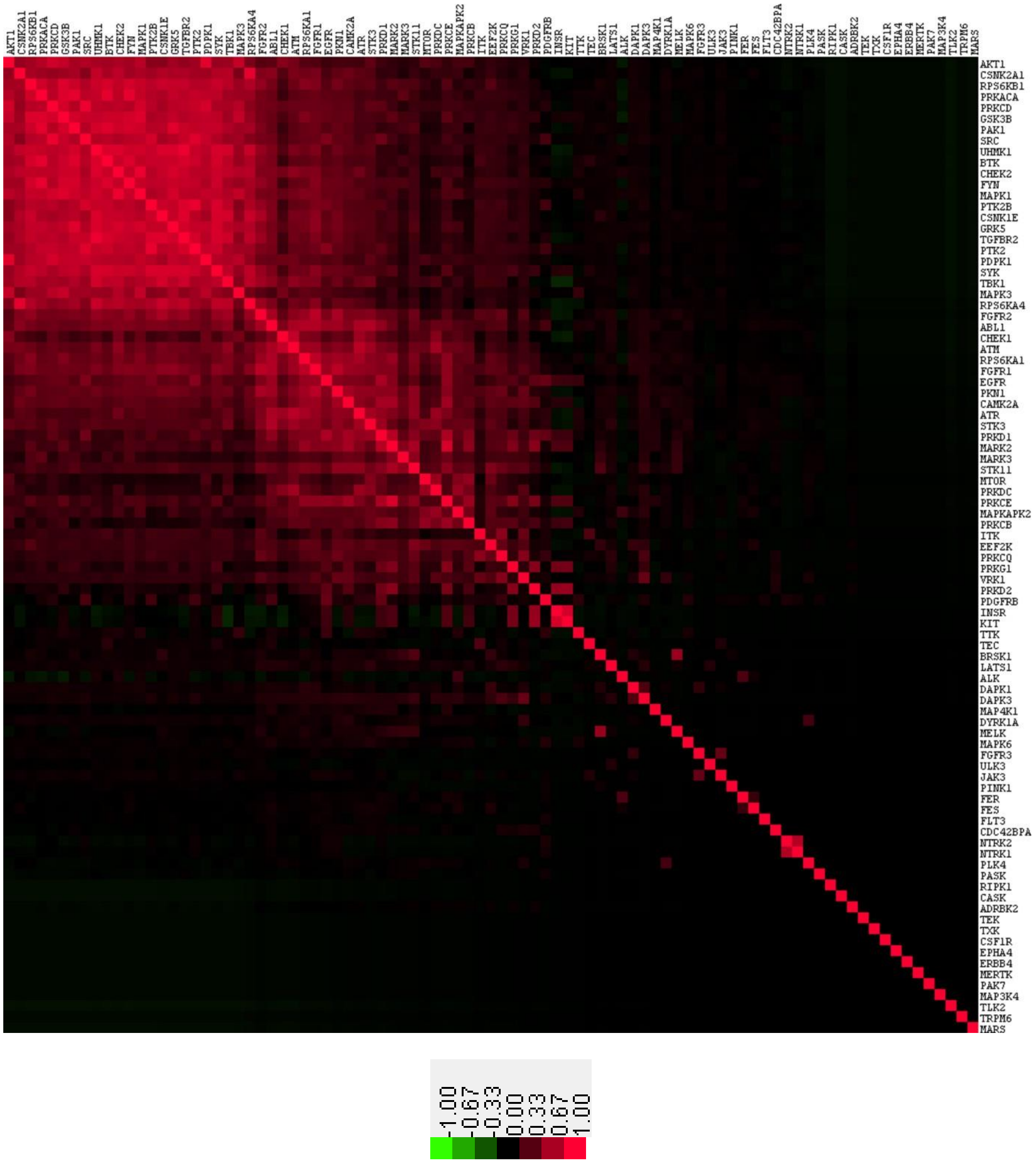

Figure S2: The first-level network module of the kinase-substrate interaction network (KSIN) and heat map of module correlation (similarity) matrices identified by the ModuLand algorithm [1, 2]. (**A**) The first-level network module of the KSIN. Each of the meta-nodes represents a module, and its name identifies the module central node. (**B**) The heat map of module correlation (similarity) matrices. The high correlation denotes that two modules are similar.

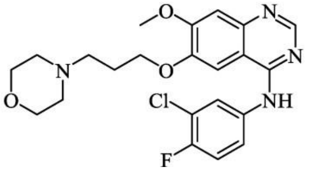

Gefitinib (DB00317)
Target: EGFR
Clinical use: NSCLC

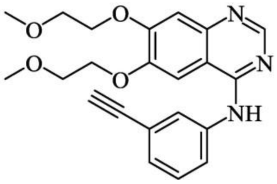

Erlotinib (DB00530)
Target: EGFR
Clinical use: NSCLC

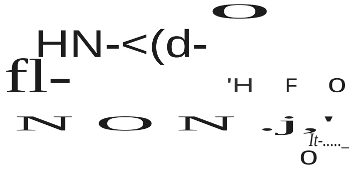

Lapatinib (DB01259)
Target: EGFR, ERBB2
Clinical use: BC

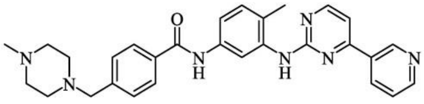

lmatinib (DB00619)
Target: ABL, KIT, PDGFR
clinical use: CML, GIST

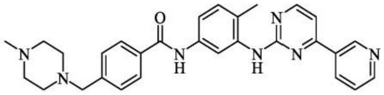

Dasatinib (DB01254)
Target: ABL, SRC, KIT, PDGFR
Clinical use: CML

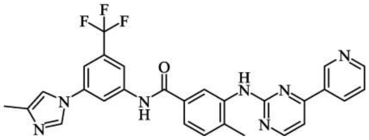

Nilotinib (DB04868)
Target: ABL
Clinical use: CML

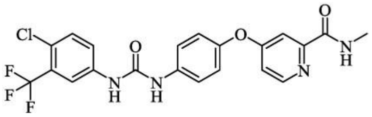

Sorafenib (DB00398)
Target: RAF, PDGF
Clinical use: RCC

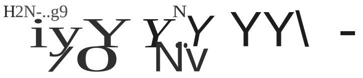

Pazopanib (DB06589)
Target: VEGFR, PDGFRA, PDGFRB, KIT
Clinical use: RCC

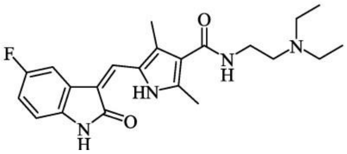

Sunitinib (DB01268)
Target: PDGFRA, PDGFRB, FLT3, VEGFR(KDR), KIT,
Clinical use: RCC, GIST

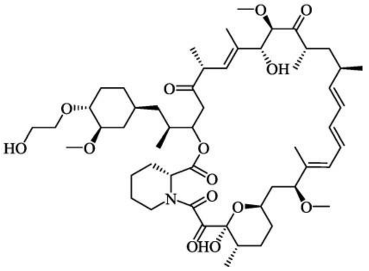

Everolimus (DB01590)
Target: MTOR
Clinical use: BC

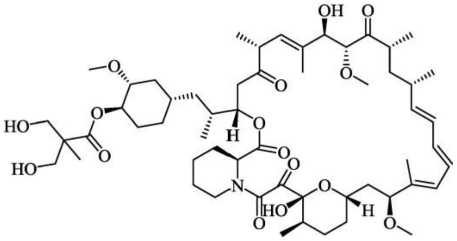

Temsirolimus (DB06287)
Target: MTOR
Clinical use: RCC

Figure S3: The chemical structure, target proteins, and FDA approved clinical usages of 11 molecularly targeted kinase inhibitors.

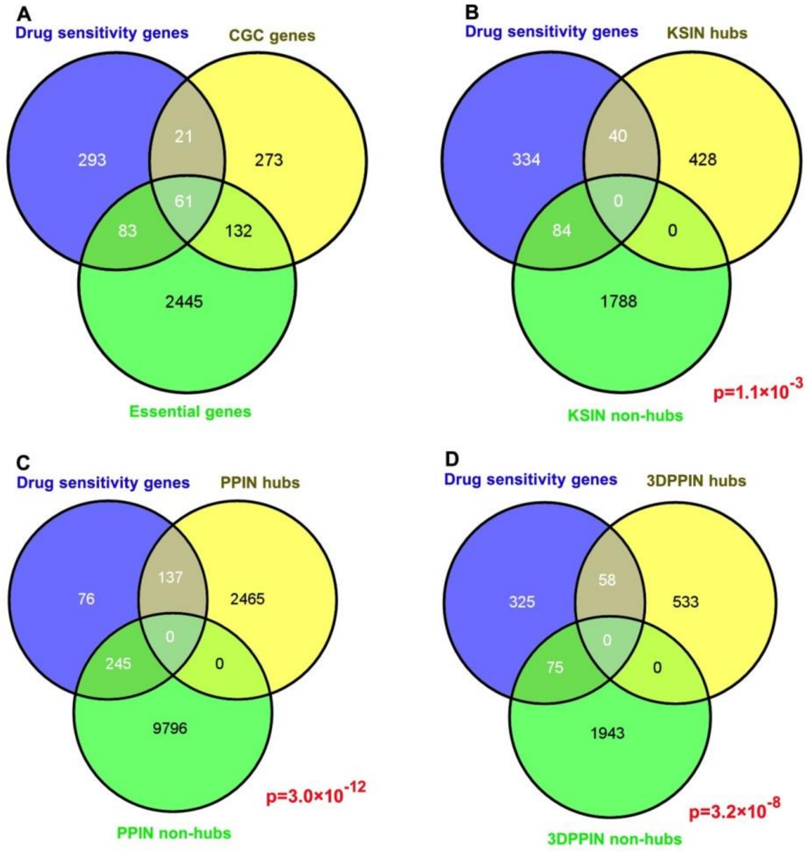

Figure S4: The Venn diagram of overlaps between hubs versus non-hubs in the protein interactome for drug response genes. (**A**) The overlap of drug resistance or sensitivity genes, Cancer Gene Census (CGC) genes, and essential genes. (**B**) The overlap of drug resistance genes in kinase-substrate interaction network (KSIN) hubs *versus* non-hubs. (**C**) The overlap of drug resistance genes in protein-protein interaction network (PPIN) hubs *versus* non-hubs. (**D**) The overlap of drug resistance genes in three-dimensional structural protein-protein interaction network (3DPPIN) hubs *versus* non-hubs.

REFERENCES

1. Szalay-Beko M, Palotai R, Szappanos B, Kovacs IA, Papp B, Csermely P. ModuLand plug-in for Cytoscape: determination of hierarchical layers of overlapping network modules and community centrality. Bioinformatics. 2012; 28: 2202-2204.
2. Kovacs IA, Palotai R, Szalay MS, Csermely P. Community landscapes: an integrative approach to determine overlapping network module hierarchy, identify key nodes and predict network dynamics. PLoS One. 2010; 5: e12528.
